# BOSC 2022: the first hybrid and 23rd annual Bioinformatics Open Source Conference

**DOI:** 10.12688/f1000research.125043.1

**Published:** 2022-09-12

**Authors:** Nomi L. Harris, Karsten Hokamp, Hervé Ménager, Monica Munoz-Torres, Deepak Unni, Nicole Vasilevsky, Jason Williams

**Affiliations:** 1 Lawrence Berkeley National Laboratory, Berkeley, CA, 94720, USA; 2 Smurfit Institute of Genetics, Trinity College Dublin, Dublin, Ireland; 3 Institut Pasteur, Paris, France; 4 University of Colorado Anschutz Medical Campus, Aurora, CO, USA; 5 Swiss Institute of Bioinformatics, Basel, Switzerland; 6 Cold Spring Harbor Laboratory, Cold Spring Harbor, NY, USA

**Keywords:** bioinformatics; open source; open science; Diversity, Equity and Inclusion

## Abstract

The 23 ^rd^ annual Bioinformatics Open Source Conference (BOSC 2022) was part of this year’s conference on Intelligent Systems for Molecular Biology (ISMB). Launched in 2000 and held every year since, BOSC is the premier meeting covering open source bioinformatics and open science. ISMB 2022 was, for the first time, a hybrid conference, with the in-person component hosted in Madison, Wisconsin (USA). About 1000 people attended ISMB 2022 in person, with another 800 online. Approximately 200 people participated in BOSC sessions, which included 28 talks chosen from submitted abstracts, 46 posters, and a panel discussion, “Building and Sustaining Inclusive Open Science Communities”. BOSC 2022 included joint keynotes with two other COSIs. Jason Williams gave a BOSC / Education COSI keynote entitled "Riding the bicycle: Including all scientists on a path to excellence". A joint session with Bio-Ontologies featured a keynote by Melissa Haendel, “The open data highway: turbo-boosting translational traffic with ontologies.”

## Introduction

**Figure 1.**
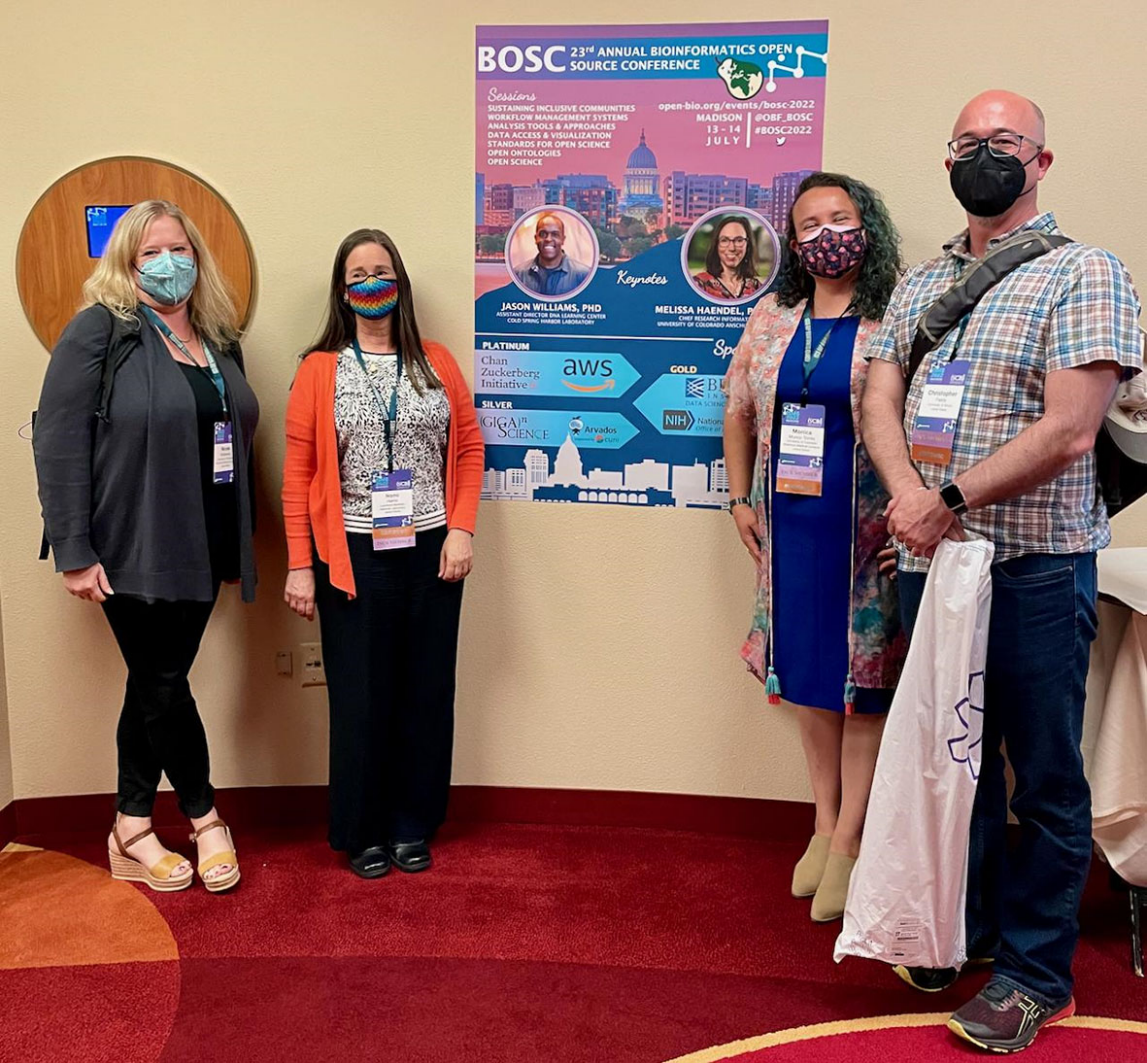
Some of the BOSC organizers (Nicole Vasilevsky, Nomi Harris, and Monica Munoz-Torres) with OBF Secretary Chris Fields. Other BOSC organizing committee members not shown: Jason Williams, Deepak Unni, Hervé Ménager, Karsten Hokamp. Shared under a CC-BY-SA license.

The 23 ^rd^ annual Bioinformatics Open Source Conference, BOSC 2022, was held as a Community of Special Interest, or COSI, which are tracks of the conference on Intelligent Systems in Molecular Biology, ISMB 2022. Occurring annually since 2000, BOSC has been part of ISMB in all but two occasions, in 2018 and 2020, when it partnered with the Galaxy Community Conference (GCC). BOSC’s parent organization is the Open Bioinformatics Foundation (OBF), a non-profit, volunteer-run organization ( Figure 1) that promotes open source software and Open Science in the biological research community.

Similar to BOSC 2021, which included a joint session with the Function COSI, BOSC 2022 featured joint keynotes with two other COSIs at ISMB: the Education and Bio-Ontologies tracks. It also featured the return of a panel, which we had not attempted to offer during the two years (2020 and 2021) when the meeting was held virtually.

Geraldine van der Auwera spoke for many when she observed, “[BOSC] is my favorite conference because it brings together such a welcoming group of people, and the lineup of talks typically offers a great balance of technical learning and community-building insights.”

### The first hybrid BOSC

Due to the continued risk of COVID-19 worldwide, ISMB held the conference in a hybrid format, welcoming attendees in person and online via a virtual conference platform. The in-person component was hosted at the beautiful Monona Terrace Community and Convention Center (designed by Frank Lloyd Wright) in Madison, Wisconsin, USA. There were 997 in-person and 776 online participants. Overall, the conference was successful, although running a hybrid meeting remains more difficult than running an all in-person or all online event. Unfortunately, some sessions were plagued with technical issues, and the interaction between in-person and online participants was minimal. Regardless, hybrid meetings are probably here to stay, given the major advantage of greater inclusivity that online meetings offer, and taking into account the financial, environmental, and public health considerations associated with in-person attendance. As we all adjust to this new hybrid format, lessons learned from this year’s experience will help improve the social and technical aspects of future hybrid meetings.

## Diversity, equity and inclusion

As in previous years, BOSC prioritized its time and funds to ensure our meeting was more diverse and inclusive. With help from our sponsors, we granted free registration to 18 participants, including 16 attendees from groups that are underrepresented in bioinformatics, and covered travel expenses for several of the panelists and keynote speakers. Diversity, Equity and Inclusion was a focus of several of the BOSC sessions, including the panel and the session on Inclusion & Open Science (see below for details).

**Figure 2.**
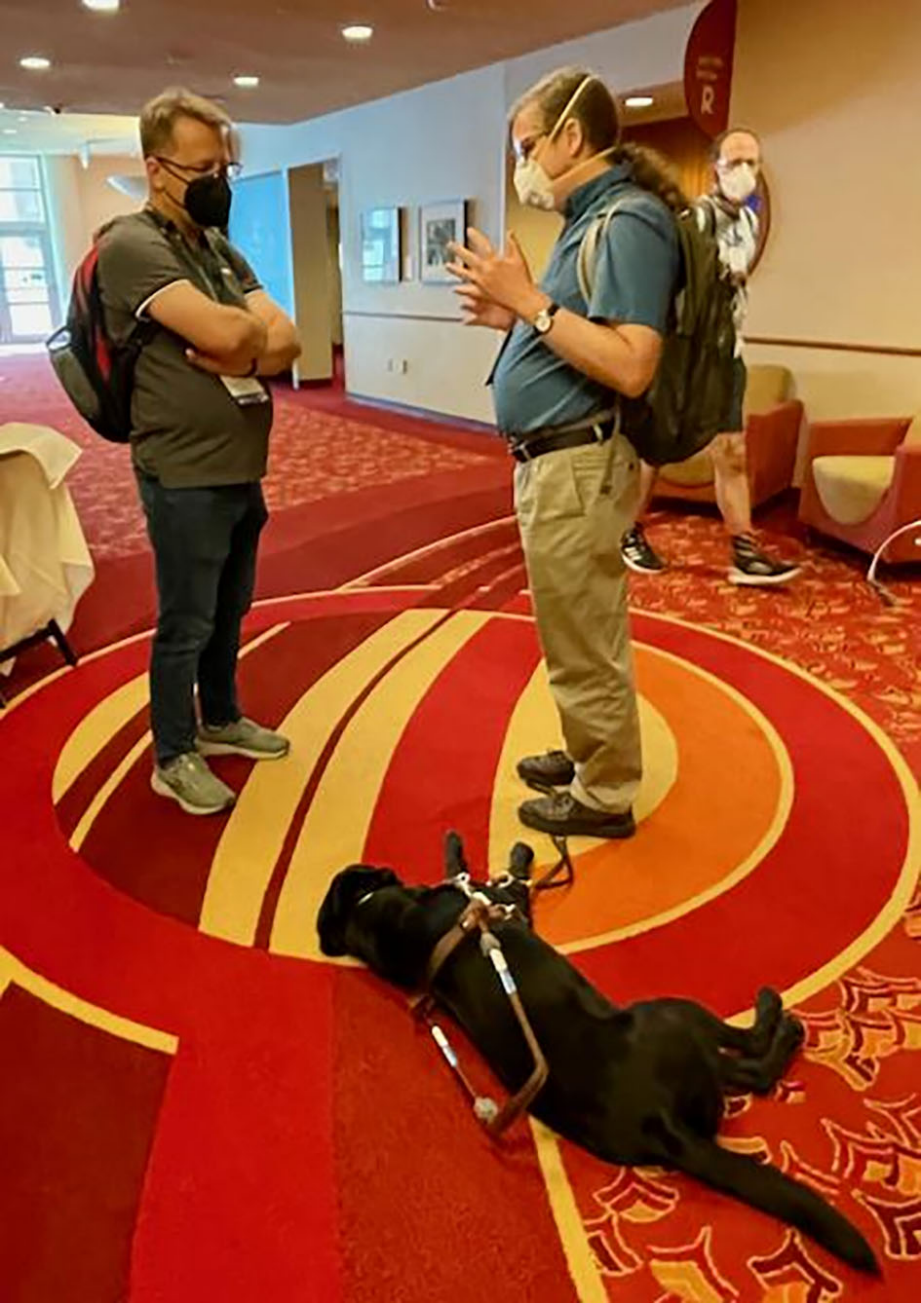
BOSC participants Scott Cain and Drew Hasley chatted while Drew’s service dog, Shade, rested at his feet. Shared under a CC-BY-SA license.

As far as we are aware, BOSC 2022 was the first BOSC, possibly even the first ISMB, to include a service dog ( Figure 2), demonstrating tangible progress in our efforts to make BOSC more inclusive and accessible.

## Conference program

### Keynotes

**Figure 3.**
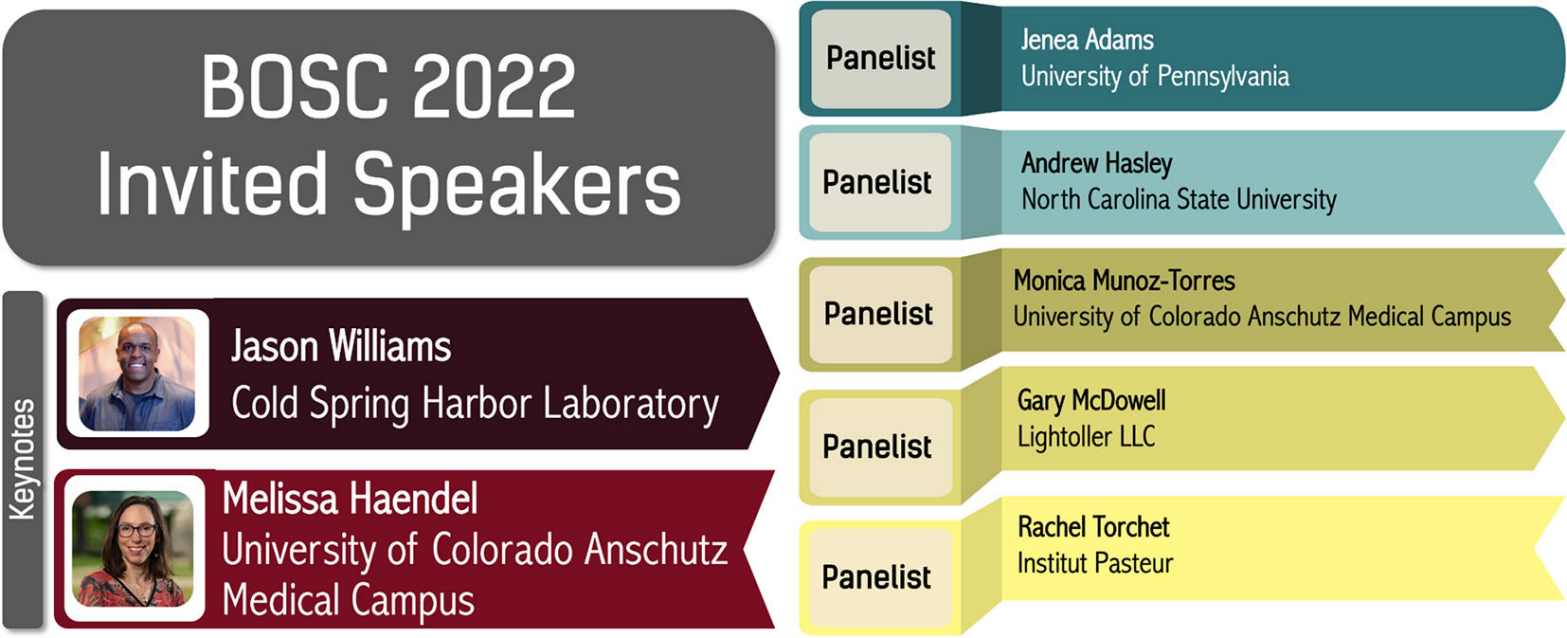
BOSC 2022 keynotes and panelists.

**Figure 4.**
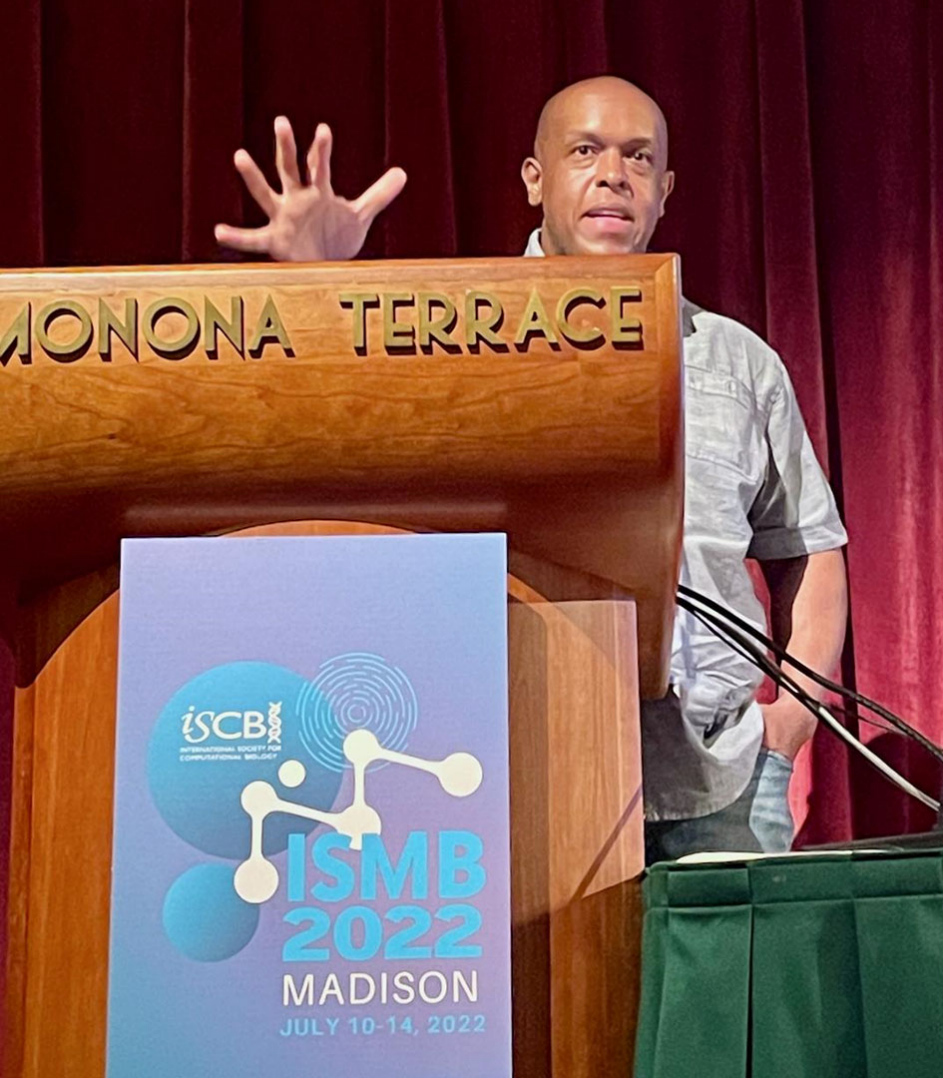
Keynote speaker Jason Williams discussed the “bicycle principles” for bioinformatics short-format training in a joint BOSC/Education COSI talk. Shared under a CC-BY-SA license.

This year’s program included joint keynotes with two other COSIs ( Figure 3). Jason Williams ( Figure 4) gave a BOSC/Education COSI keynote entitled “Riding the bicycle: Including all scientists on a path to excellence”. Jason drew on his extensive experience as an educator at the Cold Spring Harbor DNA Learning Center, discussing the newly coined “bicycle principles” for making bioinformatics short-format training more effective, persistent, and inclusive. The principles are centered around two cyclic – hence “bi-cycle” – and iterative processes (see bikeprinciples.org, where you can offer feedback on the proposed principles). One “wheel” is made up of the Core Principles, which apply to all short-format training:

• Use best evidence
• Promote catalytic learning
• Be effective; provide evidence of learning
• Be inclusive

The other “wheel” is the Community Principles, which apply when short-format training is organized by communities:

• Reach
• Scale
• Sustain

**Figure 5.**
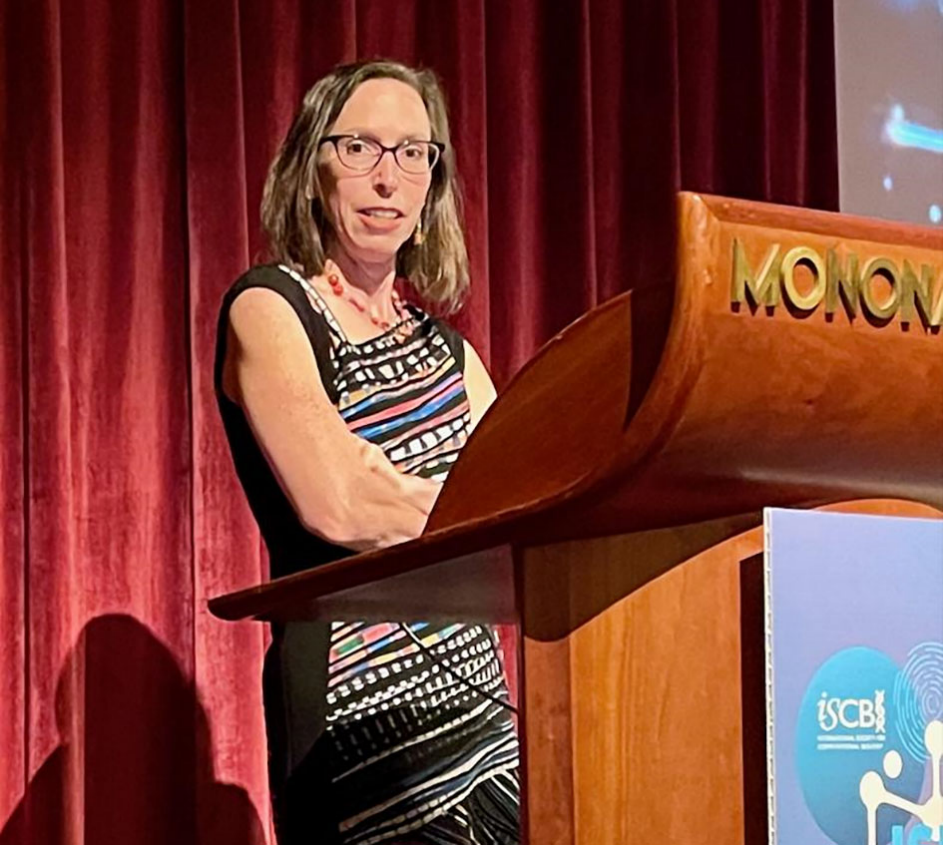
In a joint BOSC/Bio-Ontologies session, keynote speaker Melissa Haendel took attendees on a fast ride down the translational highway with the help of ontologies. Shared under a CC-BY-SA license.

The second keynote speaker, Melissa Haendel ( Figure 5), headlined a joint session with Bio-Ontologies, In her talk, “The open data highway: turbo-boosting translational traffic with ontologies.” Melissa described some of the problems involved in integrating, harmonizing, and mapping biomedical data between multiple resources and standards. For example, there are many different terminologies and definitions of diseases, so how can we tell whether two diseases discussed in different studies are in fact equivalent? Melissa leads projects such as the Mondo disease ontology, the Human Phenotype Ontology (HPO), and the LinkML data modeling language, which aim to help address those challenges. Melissa discussed ways to bridge the translational divide by leveraging semantics and ontologies — with the caveat that in most cases, if you think you need to invent a new ontology, you actually don’t.

### Panel

**Figure 6.**
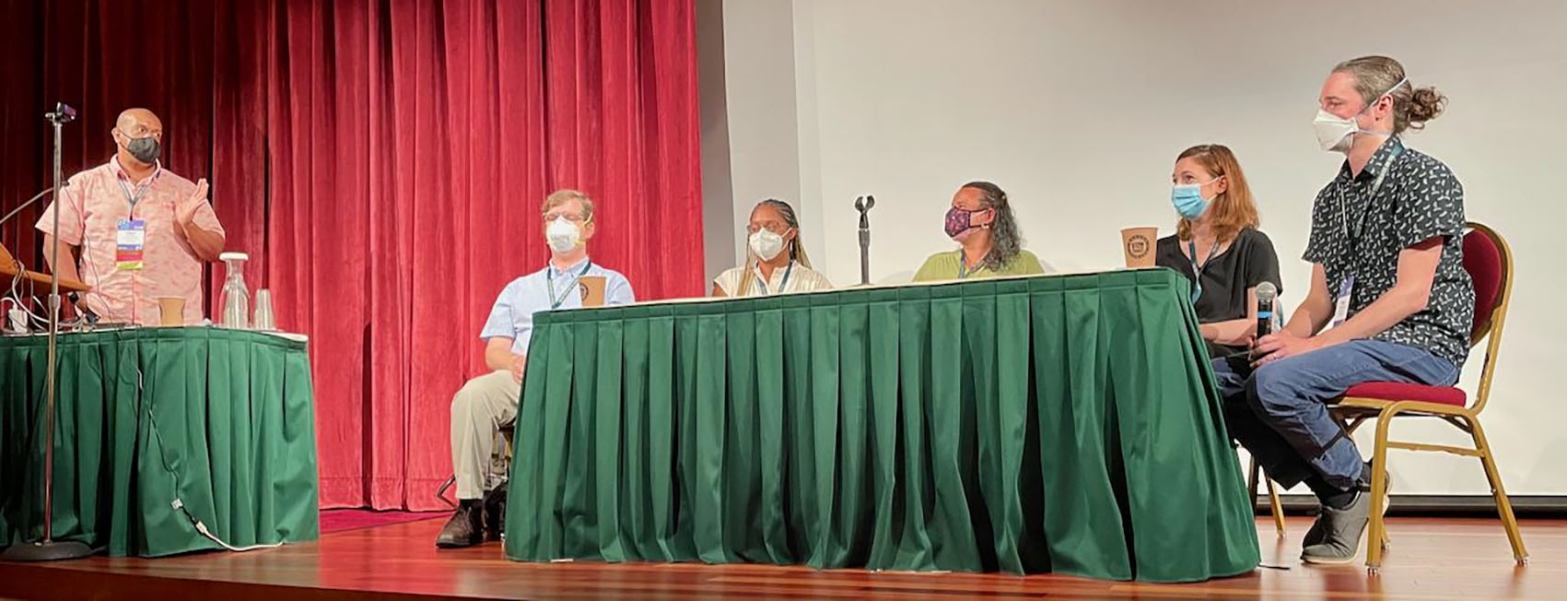
Jason Williams moderating the panel on “Building and Sustaining Inclusive Open Science Communities”. Shared under a CC-BY-SA license.

BOSC 2022 ended with a panel ( Figure 6) entitled “ Building and Sustaining Inclusive Open Science Communities” with panelists who not only are known for supporting diversity and inclusion, but also themselves are members of groups that are underrepresented in our field. As most of us are well aware, building great software is just a starting point; to maximize the impact, it’s also necessary to put effort into maintaining it. Similarly, open source and open science communities need to be maintained and expanded, and initial efforts at inclusion and diversity need to be sustained and extended. This lively, interactive panel focused on the “what next?” part -- what needs to come after the initial steps to diversify an open source project or community.

Led by Jason Williams, who is well known for his work in diversity and inclusion, the panel included Jenea Adams, founder of the Black Women in Computational Biology (BWCB) Network; Andrew Hasley, a blind biologist with an interest in Universal Design for Learning, a framework for creating inclusive instructional environments; Monica Munoz-Torres, who works to improve diversity in the bioinformatics workforce; Gary McDowell, whose focus is on early-career researchers; and Rachel Torchet, who co-founded an organization to promote diversity, equality, and inclusion at the Institut Pasteur.

The panelists discussed challenges and opportunities that we can all tackle as a community to be more inclusive. As audience member Hilmar Lapp – who, by the way, is the only person who has attended all 23 BOSCs! – observed, “The panel reminded us that we all have much more agency than we think in making spaces welcoming to everyone who wants to enter.”

### Talks and posters

As usual, BOSC included topical sessions with short and long talks chosen from submitted abstracts. The complete talk schedule can be found here. This year’s session topics were:

• Inclusion & Open Science
• Analysis tools & approaches
• Data access and visualization
• Standards for Open Science
• Workflow Management Systems
• Open ontologies and tools (joint session with Bio-Ontologies)

The new session on Inclusion & Open Science featured a talk by Rachel Torchet on gender-based disparities and biases in science, with a quantitative analysis based on observational study of the French Open Days in Biology, Computer Science and Mathematics (JOBIM for its initials in French) bioinformatics conference. In one notable finding, the online JOBIM attracted more female attendees than previous in-person conferences, but even so, women attendees asked half as many questions as men.

**Figure 7.**
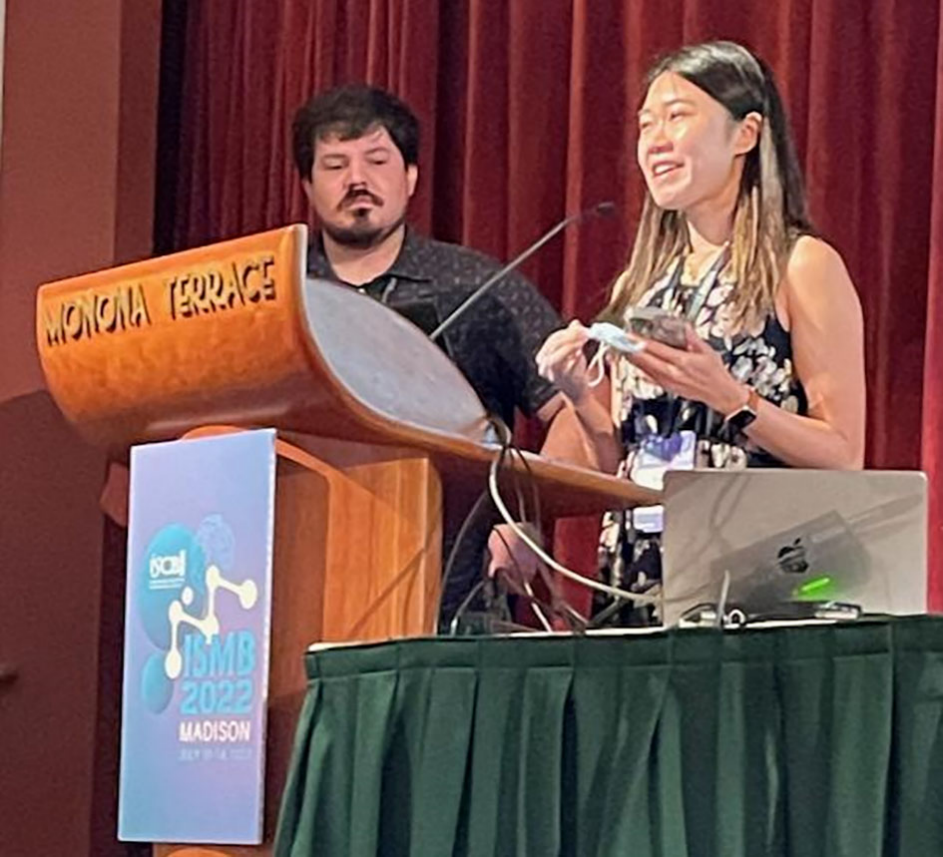
Matthew Gazzara and Farica Zhuang presented “A cloud based international community effort for reproducible benchmarking of genomic tools” during the Workflows session. Shared under a CC-BY-SA license.

**Figure 8.**
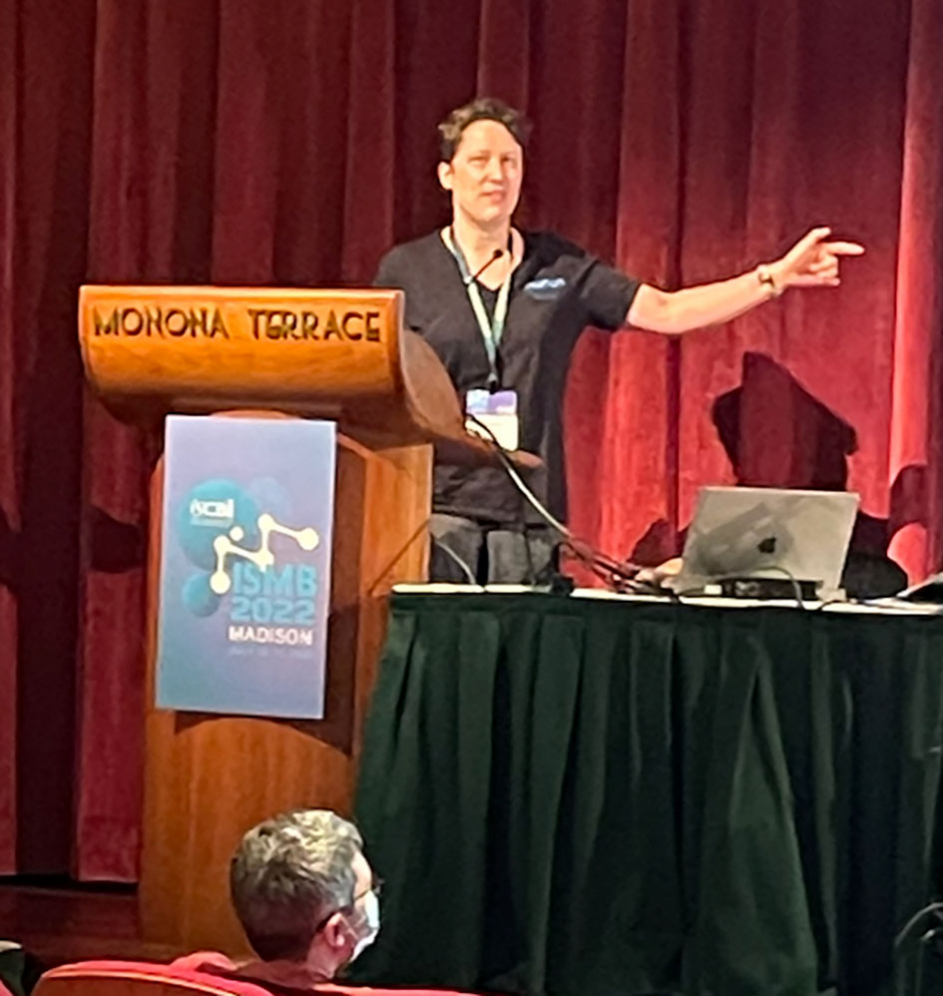
Geraldine van der Auwera heroically compressed her 20-minute talk about deciphering WDL workflows into a 5-minute lightning presentation. Shared under a CC-BY-SA license.

The always popular Workflow Management Systems session ( Figure 7) included a number of talks related to Common Workflow Language (CWL), which grew out of a BOSC CodeFest (as it was then known) in 2014. The hero of this session was Geraldine van der Auwera ( Figure 8), who compressed her 20-minute talk about deciphering Workflow Description Language (WDL) workflows into a 5-minute lightning presentation to help make up for an otherwise catastrophic delay earlier in the session due to technical issues.

**Figure 9.**
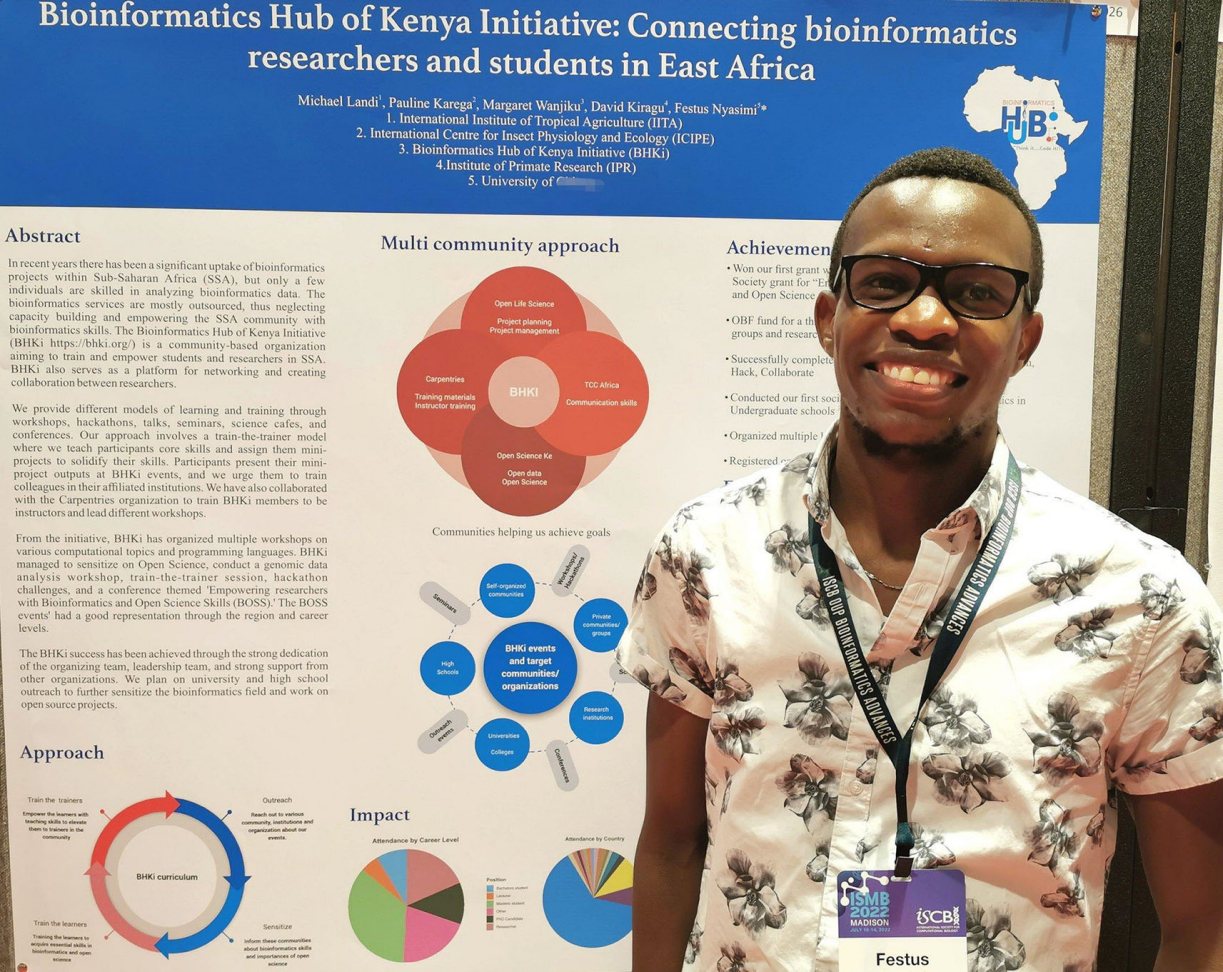
Poster presenter Festus Nyasimi, who received an OBF Event Fellowship to help defray his expenses in attending ISMB/BOSC. Shared under a CC-BY-SA license.

46 posters were presented at BOSC 2022, 29 in person ( Figure 9) and 17 in the online platform.

### CoFest

**Figure 10.**
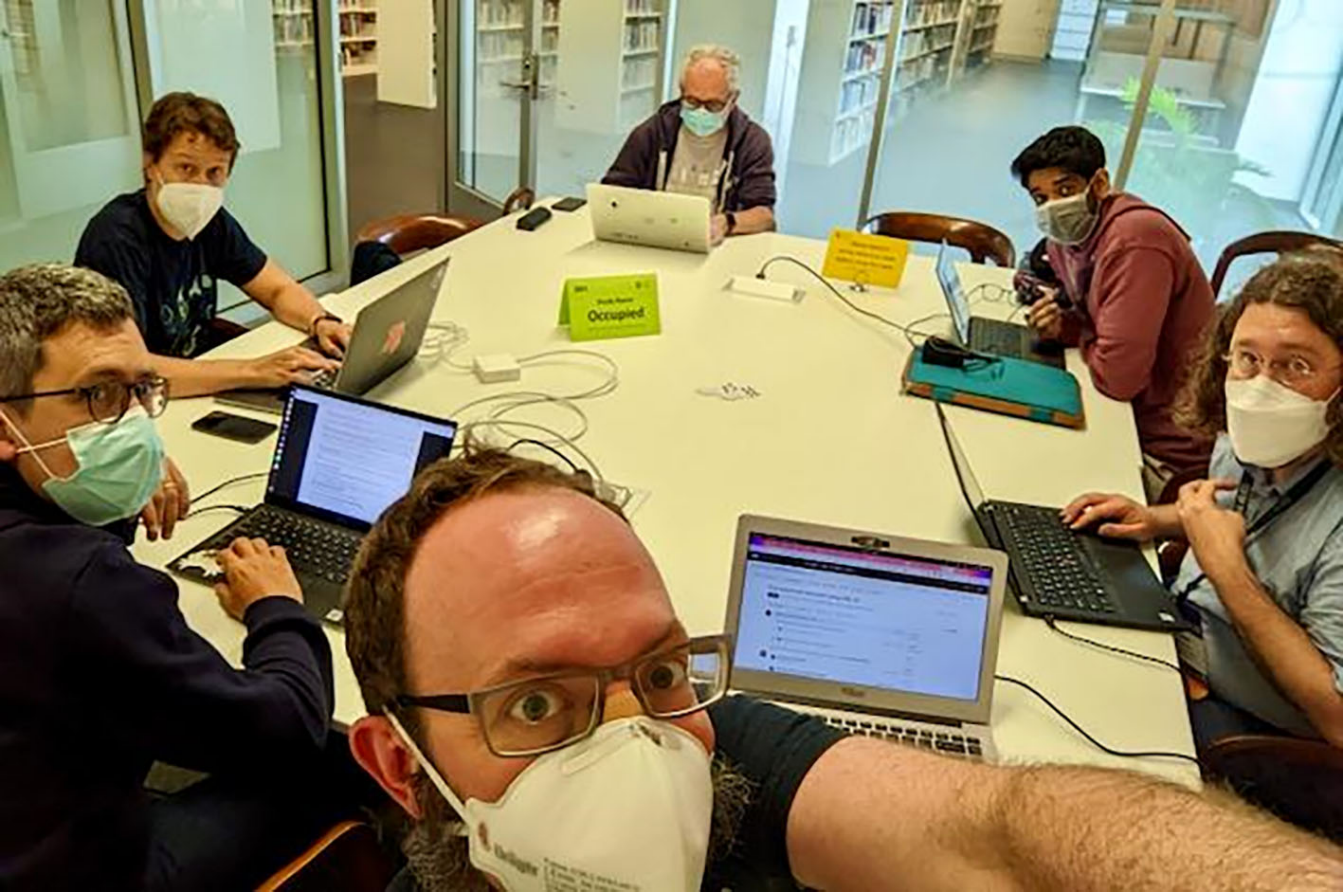
Some of the in-person CoFest 2022 participants working together at the Madison Public Library. Shared under a CC-BY-SA license.

As usual, BOSC/ISMB were followed by a two-day CollaborationFest (CoFest). A hands-on-keyboards event that has been held after BOSC for the last twelve years, CoFest was originally called CodeFest, and was renamed “CollaborationFest” in 2018 to acknowledge the importance of activities besides coding, such as working on documentation, training materials, and use cases. Like ISMB, CoFest 2022 was a hybrid meeting, with some participants in person ( Figure 10) and others online. Participants worked together on six collaborative projects, three primarily involving in-person participants and three online. Bhavesh Patel, a first-time BOSC and CoFest attendee, observed, “The CoFest was my favorite part as it provided a very informal setting to get to know all the participants on a personal level. It also gave me a unique opportunity to discuss personally with experts of the field and get one-on-one feedback on my work.”

## Data availability

No data are associated with this article.

